# Claudin-1 targeting suppresses tumor growth, invasion and metastasis in patient-derived cholangiocarcinoma models

**DOI:** 10.1126/scitranslmed.adp2595

**Published:** 2026-06-03

**Authors:** Zeina Nehme, Marion Muller, Jade Brochon, Emilie Crouchet, Frank Jühling, Mélissa Messé, Laura Heydmann, Julien Moehlin, Romain Desert, Fabio Del Zompo, Christine Thumann, Joachim Lupberger, Natascha Roehlen, Emanuele Felli, Patrick Pessaux, Aïna Venkatasamy, Patrice Marchand, Mihaela Alina Onea, Roberto Iacone, Markus Meyer, Alberto Toso, Nabeel Bardeesy, Daniela Sia, Josep M. Llovet, Yujin Hoshida, Lipika Goyal, Mirian Fernández-Vaquero, Vikas Prakash Ranvir, Mathias Heikenwälder, Tessa Ostyn, Tania Roskams, Patrice Laquerriere, Laurent Mailly, Catherine Schuster, Thomas F. Baumert

**Affiliations:** 1 University of Strasbourg, Inserm, UMR_S1110, Institute of Translational Medicine and Liver Disease (ITM); 67000 Strasbourg, France; 2 CNRS, Institut Pluridisciplinaire Hubert Curien UMR_7178; 67200 Strasbourg, France; 3 Hospital Group Saint Vincent, 67000 Strasbourg, France; 4 Unit of Hepato-Bilio-Pancreatic Surgery, Department of Visceral and Digestive Surgery, Strasbourg University Hospitals; 67000 Strasbourg, France; 5 Pôle Hépato-digestif, Strasbourg University Hospitals, 67000 Strasbourg, France; 6 IHU Strasbourg; 67000 Strasbourg, France; 7 Department of Pathology, Strasbourg University Hospital; 67200 Strasbourg, France; 8 Biological Resource Centre, Strasbourg University Hospitals; 67200 Strasbourg, France; 9 Alentis Therapeutics; 4123 Allschwil, Switzerland; 10 Center for Cancer Research, Massachusetts General Hospital; Boston, MA 02114, USA; Department of Medicine, Harvard Medical School; Boston, MA 02115, USA; 11 Mount Sinai Liver Cancer Program, Division of Liver Diseases, Department of Medicine, Tisch Cancer Institute, Icahn School of Medicine at Mount Sinai; New York, NY 10029, USA; 12 Liver Cancer Translational Research Group, Institut d’Investigacions Biomèdiques August Pi i Sunyer (IDIBAPS), Liver Unit-Hospital Clínic, Universitat de Barcelona; 08036 Barcelona, Catalonia, Spain; 13 Institució Catalana de Recerca i Estudis Avançats; 08010 Barcelona, Catalonia, Spain; 14 Liver Tumor Translational Research Program, Harold C. Simmons Comprehensive Cancer Center, Division of Digestive and Liver Diseases, University of Texas Southwestern Medical Center, Dallas; TX 75390, USA; 15 Division of Oncology, Stanford School of Medicine; Palo Alto, CA 94305, USA; 16 Division of Chronic Inflammation and Cancer, German Cancer Research Center; 69120 Heidelberg, Germany; 17 M3 Research Institute; University of Tuebingen; 72076 Tübingen, Germany; 18 Department of Imaging and Pathology, University of Leuven; 3000 Leuven, Belgium; 19 Gastroenterology-Hepatology Service, Strasbourg University Hospitals; 67000 Strasbourg, France; 20 Institut Universitaire de France; 75231 Paris, France

## Abstract

Cholangiocarcinoma (CCA) is an adenocarcinoma of the hepatobiliary system that has recently risen in incidence and mortality with unsatisfactory treatment options. Claudin-1 (CLDN1) is a transmembrane protein expressed in tight junctions and exposed on the cell surface in liver fibrosis and cancer. Using single cell RNA-sequencing (scRNA-Seq) and spatial transcriptomics of tissues of patients with CCA, we show that CLDN1 expression is upregulated in cancer cells and is associated with stemness and cell fate. Genetic gain-of-function studies in CCA orthotopic in vivo mouse models showed decreased survival and enhanced tumor growth, unraveling a functional role of CLDN1 as an oncogenic driver. Targeting exposed non-junctional CLDN1 using highly specific CLDN1 monoclonal antibodies (mAbs) inhibited tumor growth across CCA CDX and PDX mouse models and patient-derived CCA organoids, including tumors with medium or low CLDN1 expression. Moreover, antibody treatment inhibited tumor cell migration, invasion, and extrahepatic metastasis. Mechanistically, targeting exposed cell surface CLDN1 on CCA tumors using mAbs inhibited Notch1 and TROP2/STAT3 signaling pathways, resulting in decreased cancer cell stemness and EMT. Loss-of-function studies using CRISPR-Cas9 and RNAi combined with rescue and confocal imaging studies confirmed the functional and mechanistic role of these pathways. In conclusion, these results uncover CLDN1 as a previously undiscovered CCA driver and therapeutic target, paving the way for the clinical development of CLDN1 mAbs to improve the dismal outcome of patients with advanced CCA.

## Introduction

Cholangiocarcinoma (CCA) is a heterogeneous and highly aggressive adenocarcinoma of the hepatobiliary system (1). It is mainly divided into three primary anatomical subtypes, including intrahepatic CCA (iCCA), perihilar CCA (pCCA) or distal CCA (dCCA) (2). Ranking after hepatocellular carcinoma (HCC) as the second most common primary hepatic malignancy, CCA represents approximately 15% of all primary liver tumors and accounts for 2% of cancer-related deaths worldwide (3).

Chronic biliary inflammation or cholestasis are identified as major drivers of CCA initiation, suggesting a prominent role of the tumor microenvironment (4). This is coupled with the deregulation of key signal transduction pathways orchestrating cholangiocarcinogenesis, such as Notch, Hedgehog, STAT3 and TROP2 (5).

Although considered a rare cancer, CCA incidence rates have risen dramatically in the last decade (6). Moreover, CCA is characterized by a dismal prognosis with a median survival rate of less than 2 years due to late-stage diagnosis and poor treatment response (3). Standard-of-care (SoC) consists of platinum-based chemotherapies with or without checkpoint inhibitors (7–9). Targeted therapies that have shown encouraging clinical activity and have gained regulatory approval in CCA include the fibroblast growth factor receptor inhibitors pemigatinib and futibatinib and the isocitrate dehydrogenase 1 (IDH1) inhibitor ivosidenib (10–12). However, therapeutic responses are limited to a very small subset of patients, with significant adverse effects. Given the absence of satisfactory therapeutic options and the rising incidence of the disease, there is an urgent unmet medical need for additional first-line therapeutic approaches to treat CCA and improve patient outcome.

A growing body of evidence has shown deregulation of the expression of claudins in solid tumors (13, 14). Claudin-1 (CLDN1) is a transmembrane protein expressed in tight junctions (TJs), but also in a non-junctional form, for example, at the basolateral membrane of human hepatocytes, where it serves as a cell entry factor of hepatitis C virus, a major cause of hepatobiliary cancers (15). Whereas CLDN1 has been shown to be overexpressed in liver and biliary fibrosis (16, 17) and HCC (18), the functional role of CLDN1 in CCA is unknown. Using highly specific monoclonal antibodies (mAbs) targeting the extracellular loop 1 (ECL1) of exposed CLDN1 outside the TJs and a large series of patient-derived models combined with state-of-the-art transcriptomics and proteomics, we aimed to investigate the functional role of CLDN1 in CCA tumor biology and as a therapeutic target for intrahepatic and extrahepatic CCAs.

## Results

### CLDN1 is overexpressed in intrahepatic and extrahepatic CCA and is associated with oncogenic signaling and tumor stemness

**Figure 1:**
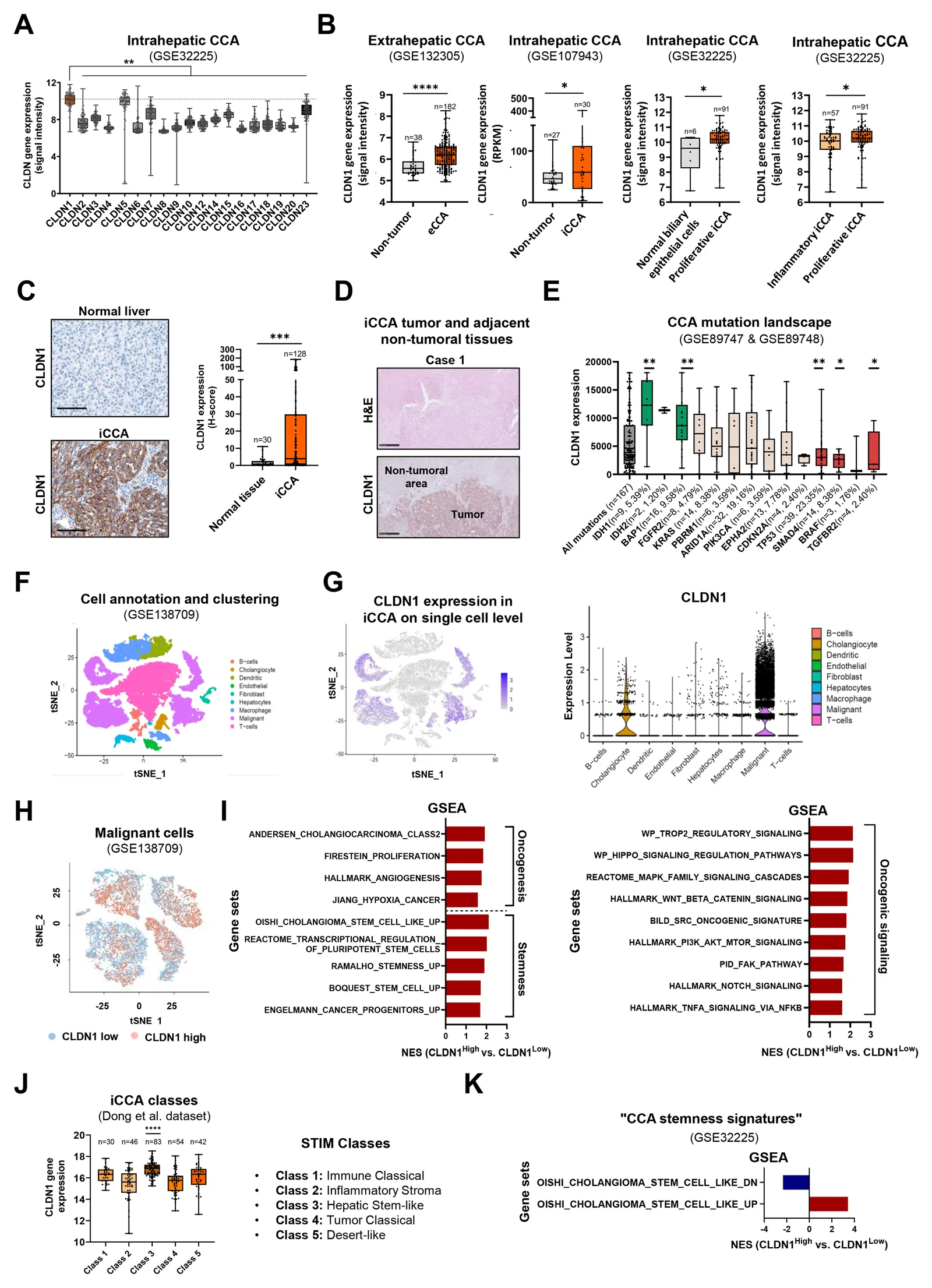
CLDN1 protein and RNA expression is upregulated in intrahepatic and extrahepatic CCA and correlates with cancer cell stemness. **(A)** Transcriptomic data of Claudin family member expression in tissues of patients with CCA (GSE132305, *n=*149, , one-way ANOVA). **(B)** Transcriptomic data of CLDN1 expression in CCA. Left panel, transcriptomic data of *CLDN1* expression in eCCA (*n=*182) compared to non-tumoral bile duct tissue (*n=*38) (GSE132305, , t-test). Middle panels, transcriptomic data of *CLDN1* expression in iCCA (*n=*30) compared to non-tumor tissues (*n=*27) (GSE107943, , t-test) and in proliferative iCCA (*n=*91) compared to non-tumoral bile duct tissues (*n=*6) (GSE32225, , Mann-Whitney test). Right panel, *CLDN1* expression in inflammatory *(n=57)* versus proliferative (*n=*91) iCCA (GSE32225, , t-test). Boxplots represent median, 1st and 3rd quartile and single data points. **(C)** CLDN1 immunostaining and quantification in healthy (*n=*30) and iCCA tissues (*n=*128) (table S1) (, Mann-Whitney test). Scale bars, 500 µm. **(D)** Representative immunostaining of CLDN1 in iCCA tissues (table S2). Scale bars, 500 µm. **(E)** Relative *CLDN1* expression in CCA tumor tissues with different genetic mutations (GSE89747 and GSE89748, , Mann-Whitney test). **(F)** Annotation of single-cell transcriptomic map (GSE138709) visualized using a t-SNE graph. Cell types were defined according to the original publication (22). **(G)** t-SNE graph (left) and violin plot (right) of *CLDN1* expression in iCCA at the single cell level. **(H)** t-SNE graph of tumor cells showing *CLDN1*^low^ or *CLDN1*^high^ expression (GSE138709). **(I)** Gene set enrichment in *CLDN1*^high^ compared to *CLDN1*^low^ tumor cells. Horizontal bars represent normalized enrichment scores (NES) of significantly altered gene sets (FDR<0.05, Kolmogorov-Smirnov test). **(J)** Transcriptomic data of *CLDN1* expression according to the STIM classification, Mann-Whitney test). **(K)** Enrichment of gene sets related to CCA stemness signatures in *CLDN1*^high^ compared to *CLDN1*^low^ tumors. Horizontal bars represent NES of significantly altered gene sets (GSE32225, FDR<0.05, Kolmogorov-Smirnov test). *p<0.05, **p ≤0.01, ***p<0.001, ****p<0.0001

To investigate the role of CLDN1 in CCA, we first analyzed its expression across different independent cohorts from Gene Expression Omnibus (GEO). Our gene expression analysis revealed that *CLDN1* is the most highly expressed Claudin family member in tissues of patients with CCA (GSE32225, **p<0.01, Fig. 1A). *CLDN1* is highly expressed in extrahepatic CCA (eCCA) (GSE132305, ****p<0.0001, Fig. 1B, left panel) and intrahepatic CCA (iCCA) (GSE107943 and GSE32225, *p<0.05, Fig. 1B, middle panels) in comparison with non-tumoral bile duct samples. In addition, *CLDN1* expression is significantly higher in proliferative compared to inflammatory iCCA (GSE32225, *p<0.05, Fig. 1B, right panel), a main biological iCCA class associated with worse patient outcome (19). Corroborating a pathological role in CCA, CLDN1 immunostaining in tissues of patients with iCCA showed a significant upregulation in tumors compared to healthy tissues (***p<0.001, Fig. 1C, table S1). This was further reflected by the high expression of CLDN1 in iCCA tumors (Fig. 1D, fig. S1A and table S2). As iCCA and eCCA display typical patterns of actionable genomic alterations that offer the opportunity for therapeutic intervention (20), we assessed *CLDN1* expression in tumors with known genetic alteration. *CLDN1* expression was significantly upregulated in tumors containing *IDH1* and *BRCA1 associated protein-1* (*BAP1)* mutations (GSE89747 and GSE89748, **p<0.01, Fig. 1E), the latter known to be more frequent in the CCA *hepatic stem-like* class described to be enriched with stem cell features (21). On the other hand, *tumor protein P53* (*TP53), mothers against decapentaplegic homolog 4* (*SMAD4)* and *transforming growth factor beta receptor 2 (TGFBR2)* mutations were associated with lower *CLDN1* expression (GSE89747 and GSE89748, *p<0.05, **p<0.01, Fig. 1E).

CCA is characterized by a strong molecular inter-tumor heterogeneity with several well-described molecular phenotypes (22). Analysis of *CLDN1* expression in human iCCA single-cell RNA-sequencing (scRNA-Seq) data revealed malignant tumor cells as the key cell population with robust *CLDN1* expression (GSE138709, Fig. 1F and G). Gene set enrichment analysis (GSEA) revealed that tumor cells highly expressing *CLDN1* (Fig. 1H) are enriched in signatures suggestive of enhanced cancer hallmarks, stemness, and oncogenic signaling (GSE138709, FDR<0.05, Fig. 1I). Similar findings were obtained in two independent cohorts of patients with CCA (GSE151530, HRA000863, FDR<0.05, fig. S1B and C). Further corroborating this association, we found that *CLDN1* is highly expressed in the molecular iCCA *hepatic stem-like* class according to Martin-Serrano et al. “Stroma, Tumour and Immune Microenvironment” (STIM) classification (21) in several cohorts (23–25) (***p<0.001, ****p<0.0001, Fig. 1J, fig. S1D and E). We also found a significant enrichment of *CLDN1* in CCA stem cell signatures in patient tumors (GSE32225, FDR<0.05, Fig. 1K), suggesting a correlation between CLDN1 and cancer stemness. Finally, high *CLDN1* expression was associated with significantly worsened survival in patients with CCA (*p<0.05, fig. S1F).

**Figure 2:**
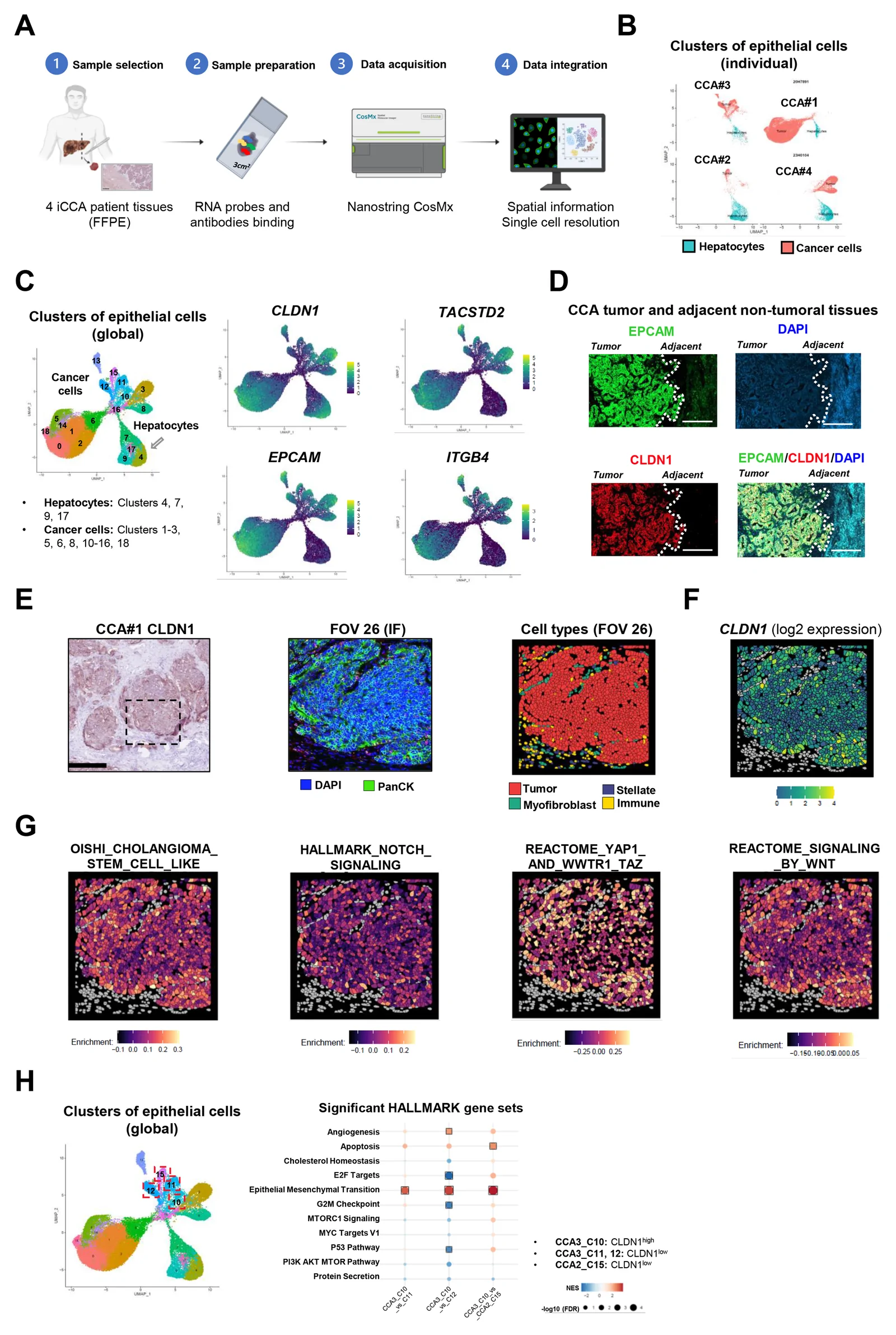
CLDN1 expression correlates with stemness markers and metastasis signatures in CCA architecture using spatial transcriptomics (ST). **(A)** Workflow of patient samples acquisition and processing strategy. **(B)** UMAP plot embedding of spatial transcriptomic spot distribution of epithelial cells colored by their cellular type (cancer cells or hepatocytes) based on the expression of marker genes from four different patients with CCA (Case 7 (CCA#1), Case 8 (CCA#2), Case 9 (CCA#3), and Case 5 (CCA#4)) (table S2). **(C)** UMAP plots of cell type enrichment from all patient clusters and *CLDN1, EPCAM, TACSTD2* and *ITGB4* expression. **(D)** Staining of EPCAM and CLDN1 by immunofluorescence in tissues of patients with iCCA (table S4). Scale bars, 100 µm. **(E)** Representative CLDN1 immunostaining used for pathological sampling, the corresponding IF staining, and the spatial map illustrating the distribution of main cell types including tumor cells, myofibroblasts, stellate, and immune cells (Field of view 26, patient CCA#1) (table S3). Scale bars, 250 µm. **(F)** *CLDN1* spatial expression in the ST map region indicated in panel E. **(G)** Enrichment of stemness and signaling pathways signatures in the corresponding spatial areas of tumor tissue and non-tumoral adjacent tissue (Field of view 26, patient CCA#1). **(H)** Dot-plot representing significant HALLMARK gene sets differentially enriched in spatial transcriptomics in the clusters *CLDN1*^high^ (CCA#3_C10) vs *CLDN1*^low^ (CCA#3_C11, C12 and CCA#2_C15). Black boxes are shown around the point when the GSEA FDR is <0.05. FOV: Field of View.

Next, we assessed *CLDN1* expression by spatial transcriptomics in tissues of four different patients with iCCA at the single cell level (Fig. 2A and B, tables S2 and 3). As shown on the clusters of epithelial cells, *CLDN1* was co-expressed with the *epithelial cellular adhesion molecule* (*EPCAM)* and the *tumor-associated calcium signal transducer 2* (*TACSTD2*/TROP2) in cancer cells (Fig. 2C), both known as key regulators of cell proliferation, fate and self-renewal (26). This was further confirmed by CLDN1 and EPCAM colocalization *in situ* in tumor tissues of patients with iCCA (Fig. 2D, table S4). Furthermore, we found that *CLDN1* was co-expressed with *integrin-β4 (ITGβ4)*, a well-established mediator of CCA metastasis during tumor progression (27) (Fig. 2C). To investigate the phenotype of *CLDN1*^+^ clusters within the CCA transcriptomic landscape, we performed a GSEA showing that *CLDN1* expression andspatial distribution (Fig. 2E and F) co-localized with stemness and key oncogenic signaling pathways, such as *Notch1, YAP/TAZ* and *Wnt* signaling (Fig. 2G, fig. S2A to E). Finally, we assessed the enriched pathways in *CLDN1*^high^ cancer cell clusters compared to *CLDN1*^low^ cell clusters. We observed that high expression of *CLDN1* is robustly associated with epithelial to mesenchymal transition (EMT) pathway activation (FDR<0.05, Fig. 2H) in CCA tumors with metastatic profiles (CCA#2 and CCA#3, table S2). Taken together, the upregulation of CLDN1 expression in CCA and its association with different functional signatures in scRNA-Seq and spatial transcriptomics, cancer stemness and metastasis, suggest a functional role of CLDN1 in CCA pathogenesis.

### CLDN1 drives carcinogenesis in vivo

**Figure 3:**
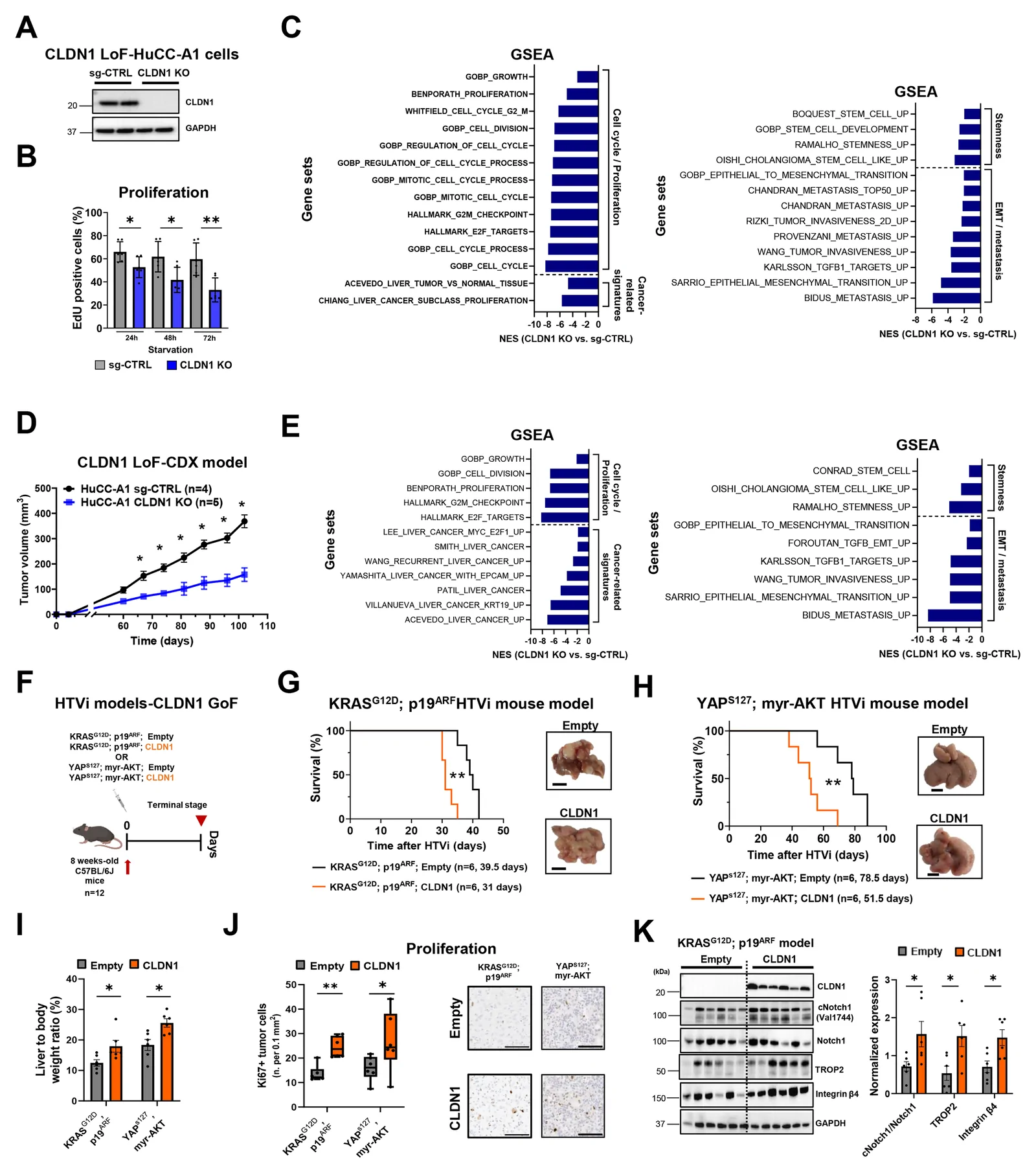
Genetic loss- and gain-of function studies reveal CLDN1 as a CCA mediator. **(A-E)** CLDN1 loss-of-function studies in human CCA models **(A)** Representative Immunoblot expression of CLDN1 and GADPH in HuCC-A1 sg-CTRL or CLDN1 knockout (KO) cells. **(B)** Inhibition of cell proliferation in HuCC-A1 CLDN1 KO vs sg-CTRL cells as shown by percentage of EdU positive cells after 24 h incubation with EdU (*N=*3, duplicate per condition, , Mann-Whitney test). Data are shown as Mean ± SD. **(C)** Gene set enrichment analysis of bulk RNASeq derived from CLDN1 KO HuCC-A1 cells compared to sg-CTRL HuCC-A1 cells. Horizontal bars represent normalized enrichment scores (NES) of significantly altered gene sets (FDR<0.05, Kolmogorov-Smirnov test). **(D)** Tumor growth in CLDN1 KO compared to sg-CTRL cells in the HuCC-A1 loss-of-function male CDX model (*n=4-*5 mice/group, , Mann-Whitney test) Data are shown as Mean ± SEM. **(E)** Gene set enrichment in CLDN1 KO compared to sg-CTRL HuCC-A1 CDX tumors. Horizontal bars represent normalized enrichment scores (NES) of significantly altered gene sets (FDR<0.05, Kolmogorov-Smirnov test). **(F-K)** CLDN1 gain-of-function studies in a CCA orthotopic immunocompetent mouse model. (**F**) Experimental approach of CLDN1 gain-of-function studies in murine models carrying either the frequently mutated driver gene KRAS in combination with loss of the tumor suppressor p19 (KRAS^G12D^; p19^ARF^ model) or dysregulation of signaling pathways known to play a crucial role in the molecular pathogenesis of iCCA including YAP1 and AKT1 **(**YAP^S127^; myr-AKT model) using hydrodynamic tail vein injection (HTVi). **(G, H left)** Survival curve of C57BL/6J male mice injected with KRAS^G12D^; p19^ARF^; Empty or KRAS^G12D^; p19^ARF^; CLDN1 (*n=*6 mice/group, , Log-rank Mantel-Cox test) (F) or with YAP^S127^; myr-AKT; Empty and YAP^S127^; myr-AKT; CLDN1 (*n=*6 mice/group,, Log-rank Mantel-Cox test). **(G, H right)** Representative pictures of livers from ventral view. Scale bars, 1 cm. **(I)** Liver to body weight ratio percentage (*p<0.05, two-sided unpaired t-test)**. (J)** Quantification and immunohistochemistry of Ki67 expression in tumor tissues (*n=*6 mice/group, , **p<0.01, Mann-Whitney test). Scale bars, 100 µm. **(K)** Representative Immunoblots and normalized expression of CLDN1, cleaved Notch1 (Val1744), Notch1, TROP2, integrin β4 and GADPH in KRAS^G12D^; p19^ARF^ model (*n=*6 mice/group, , Mann-Whitney test). Data are shown as Mean ± SEM. *p<0.05, **p<0.01

To investigate whether CLDN1 overexpression and associated perturbations in cancer cell fate and signaling observed in patient cancer tissues have a functional role in CCA biology, we performed loss-of-function studies using CRISPR/Cas9-mediated CLDN1 knock-out (KO) in human CCA cell-based and CDX models (Fig. 3A, fig. S10A). CLDN1 KO in the patient-derived iCCA cell line HuCC-A1 led to a significant and robust reduction in cell proliferation (*p<0.05, **p<0.01, Fig. 3B, fig. S3A). GSEA of the cancer cell transcriptome revealed downregulation of signatures associated with cell cycle progression, proliferation, stemness, EMT and metastasis in CLDN1 KO versus control cells (FDR<0.05, Fig. 3C). Similarly, CLDN1 KO in vivo significantly decreased tumor growth in the corresponding human CDX mouse model (Fig. 3D), accompanied by similar changes in the tumor transcriptome as shown by GSEA signatures (FDR<0.05, Fig. 3E). Reduced tumor growth in vivo and similar associations of CLDN1 expression with stemness and cancer cell fate in patient tissues and genetic loss-of-function studies, suggest that CLDN1 has a functional role in human CCA cancer cell biology.

To further investigate the functional role of CLDN1 as a CCA driver, we took advantage of well-established genetic mouse models that reliably reproduce the histologic features of human CCA (21) through hydrodynamic tail-vein injections of KRAS^G12D^; p19^ARF^ or YAP^S127^; myr-AKT expression plasmids in combination with a plasmid to overexpress CLDN1 or a control plasmid (Fig. 3F). As expected, mice from both models developed hepatobiliary tumors (fig. S3B), with mice overexpressing CLDN1 demonstrating a significant shorter overall survival and increased tumor burden compared to the control group as indicated by the liver-to-body weight ratio (*p<0.05, **p<0.01, Fig. 3G to I). Moreover, CLDN1 gain-of-function in mice with CLDN1 overexpression significantly enhanced intratumoral proliferation in both models as measured by the increase in Ki67^+^ tumor cells (*p<0.05, **p<0.01, Fig. 3J). Mechanistically, the KRAS^G12D^; p19^ARF^ and YAP^S127^; myr-AKT models represent different molecular CCA subtypes with distinct oncogenic pathways activation, actionable genotype-immunophenotypes and tumor microenvironments (28). CLDN1 overexpression in the KRAS^G12D^; p19^ARF^ model significantly enhanced Notch1 and TROP2 signaling, both implicated in stemness, as well as ITGβ4 expression (*p<0.05, Fig. 3K, fig. S10B), which is associated with shorter survival in patients with CCA (27, 29, 30). In the YAP^S127^; myr-AKT model, CLDN1 overexpression resulted in increased SRC and NF-kB signaling (*p<0.05, fig. S3C), all well-described active oncogenic pathways in CCA pathogenesis (31, 32). Overall, these *in vivo* loss- and gain-of-function studies in human and mouse CCA models suggest a functional role of CLDN1 as an oncogenic driver in CCA.

### Perturbation studies across multiple cell line-derived xenograft (CDX) and patient-derived xenograft (PDX) mouse models demonstrate CLDN1 as a CCA therapeutic target

**Figure 4:**
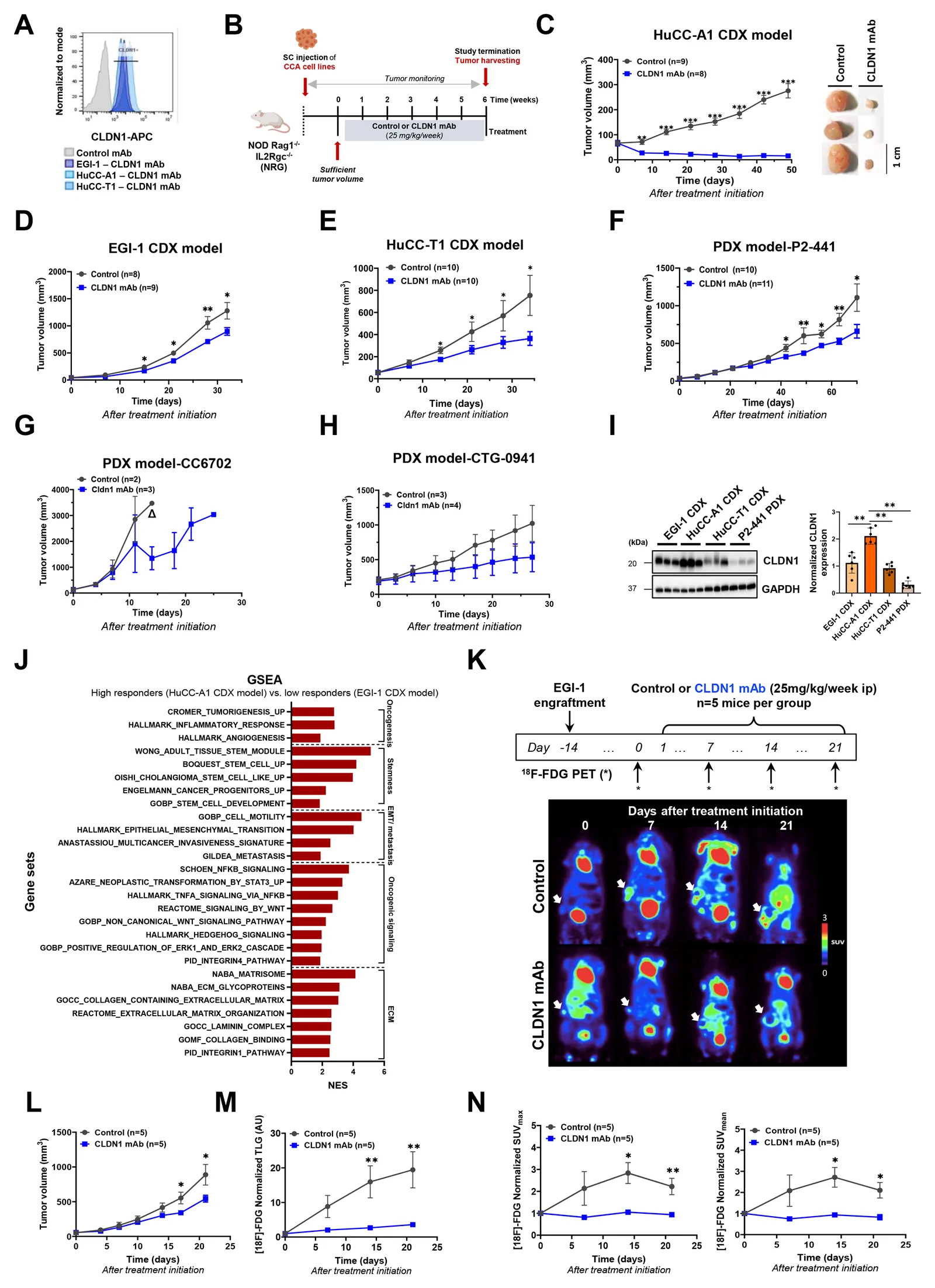
Treatment with CLDN1 mAb inhibits tumor growth in CCA CDX and PDX models and reduces CCA glucose metabolism in vivo. **(A)** Expression of cell surface CLDN1 by flow cytometry in EGI-1, HuCC-A1 and HuCC-T1 cells using an anti-CLDN1 or isotype control mAbs (*N=*3, triplicates/cell line). **(B)** Experimental approach of CDX mice model treated with CLDN1 mAbs. **(C-E)** CDX mouse models. Tumor growth in vehicle control- or CLDN1 mAb-treated in the HuCC-A1 CDX mice (*n=*8-9 mice/group, , Mann-Whitney test) and representative macroscopic images of harvested tumors (day 49) (C), the EGI-1 CDX model (*n=*8-9 mice/group, , Mann-Whitney test) (D), and the HuCC-T1 CDX model (*n=*10 mice/group, , Mann-Whitney test) (E). Data are shown as Mean ± SEM. **(F-H)** PDX mouse models. Tumor growth in vehicle control- or CLDN1 mAb-treated mice in the PDX models P2-441 (*n=*10-11 mice/group, , Mann-Whitney test) (F), CC6702 (*n=*2-3 mice/group) (G) and CTG-0941 (*n=*3-4 mice/group) (H) (table S5). Δ indicates that mice were sacrificed for reaching the terminal sacrifice endpoint. Data are shown as Mean ± SEM. **(I)** Representative Immunoblots and normalized expression of CLDN1 in tumors derived from the CDX models and the P2-441 PDX model (*n*=3 mice/model, , Mann-Whitney test). Data are shown as Mean ± SEM. **(J)** Gene set enrichment in tumors of high responders to CLDN1 mAb treatment (HuCC-A1 CDX model) compared low responders (EGI-1 CDX model). Horizontal bars represent NES of significantly altered gene sets (FDR<0.05, Kolmogorov-Smirnov test). **(K)** Upper panel, experimental [18F]-FDG PET scan imaging approach. Lower panel, representative images showing [18F]-FDG uptake in vehicle control- or CLDN1 mAb-treated CDX mice (*n=*5 mice/group). **(L)** Tumor growth in vehicle control- or CLDN1 mAb-treated EGI-1 CDX model (*n=*5 mice/group, , Mann-Whitney test). **(M, N)** Quantitative assessment of normalized TLG in [18F]-FDG PET scan growth (M) and quantitative assessment of normalized SUV_max_ (left panel) and SUV_mean_ (right panel) (N) in vehicle control- or CLDN1 mAb-treated EGI-1 CDX mice (*n=*5 mice/group, , Mann-Whitney test). Data are shown as Mean ± SEM. *p<0.05, **p<0.01, ***p<0.001

Given its overexpression in patients and functional role as an oncogenic driver in vivo, we investigated CLDN1 as a therapeutic target using fully humanized CLDN1 antibodies specifically targeting a conformation-dependent epitope in the ECL1 of non-junctional CLDN1 (16, 33). We assessed the effect of the CLDN1 mAb treatment on tumor growth in CDX mouse models using extrahepatic (EGI-1), and intrahepatic (HuCC-A1 and HuCC-T1) human CCA cell lines expressing CLDN1 at different levels (Fig. 4A and B). Treatment with the CLDN1 mAb significantly reduced tumor growth in all CDX models (*p<0.05, **p<0.01, ***p<0.001, Fig. 4C to E). The inhibition of tumor growth was accompanied by a trend for reduced Ki67 expression (ns, fig. S4A). Considering that molecular drivers and responses to therapeutics strongly vary between different CCA subclasses and patients (2), PDX mouse models have been shown to partially recapitulate tumoral heterogeneity and are currently one of the most widely used in vivo systems for studying cancer therapeutics and predicting clinical outcomes (34). Treatment with the CLDN1 mAb significantly inhibited tumor growth in 3 different PDX models, including a patient tumor (P2-441) with low CLDN1 protein expression (*p<0.05, **p<0.01, Fig. 4F to I, fig. S10C and table S5). Moreover, treatment with the CLDN1 mAb reduced tumor growth in the PDX model CTG-0941 resistant to the FGFR inhibitor pemigatinib although the difference did not reach significance (ns, Fig. 4H, fig. S4B). Collectively, these findings show the efficacy of the CLDN1 mAb as a single agent in patient-derived models. No significant changes in liver function tests or body weight were observed in CLDN1 mAb-treated animals (fig. S4C to D).

Since antibody-mediated inhibition of tumor growth was also observed in CCA tumors with low-level CLDN1 expression (**p<0.01, Fig. 4F to I), our in vivo proof-of-concept studies suggest therapeutic efficacy across different CLDN1 expression levels. Transcriptional analyses revealed that high responder tumors were enriched in gene signatures associated with enhanced stemness, EMT, activation of oncogenic signaling, and extracellular matrix (ECM) remodeling signatures (FDR<0.05, Fig. 4J). Computational intersection of transcriptomic factors with the human secretome revealed multiple blood-based candidate biomarkers among the pathways associated with treatment response (table S6) including the Notch1 ligand Delta-like ligand 1 (DLL1). This dataset may serve as a starting point to devise candidate biomarkers for patient stratification in clinical development.

Positron emission tomography (PET) with 2-[^18^F]-fluoro-2-deoxy-d-glucose (^18^F-FDG) is applied clinically for the study of tumor metabolism and treatment response (35). Here, we evaluated the effect of the CLDN1 mAb on cancer metabolism by performing ^18^F-FDG PET in EGI-1 CDX mice (Fig. 4K). Consistent with our previous results (Fig. 4D), treatment with the CLDN1 mAb significantly delayed tumor growth (*p<0.05, Fig. 4L). In addition, normalized Total Lesion Glycolysis (TLG), a standard parameter to evaluate tumor metabolic activity, was reduced in CLDN1 mAb-treated compared to control-treated mice (**p<0.01, Fig. 4M). After 2 weeks of CLDN1 mAb treatment, ^18^F-FDG PET scan revealed a robust reduction in normalized standardized uptake value (SUV)_mean_ and SUV_max_, corresponding to FDG uptake by malignant tissue (36) (*p<0.05, **p<0.01, Fig. 4N). Taken together, these data provide a robust preclinical in vivo proof-of-concept for CCA treatment using CLDN1-specific mAbs.

### CLDN1 is a driver of cell invasion and metastasis

**Figure 5:**
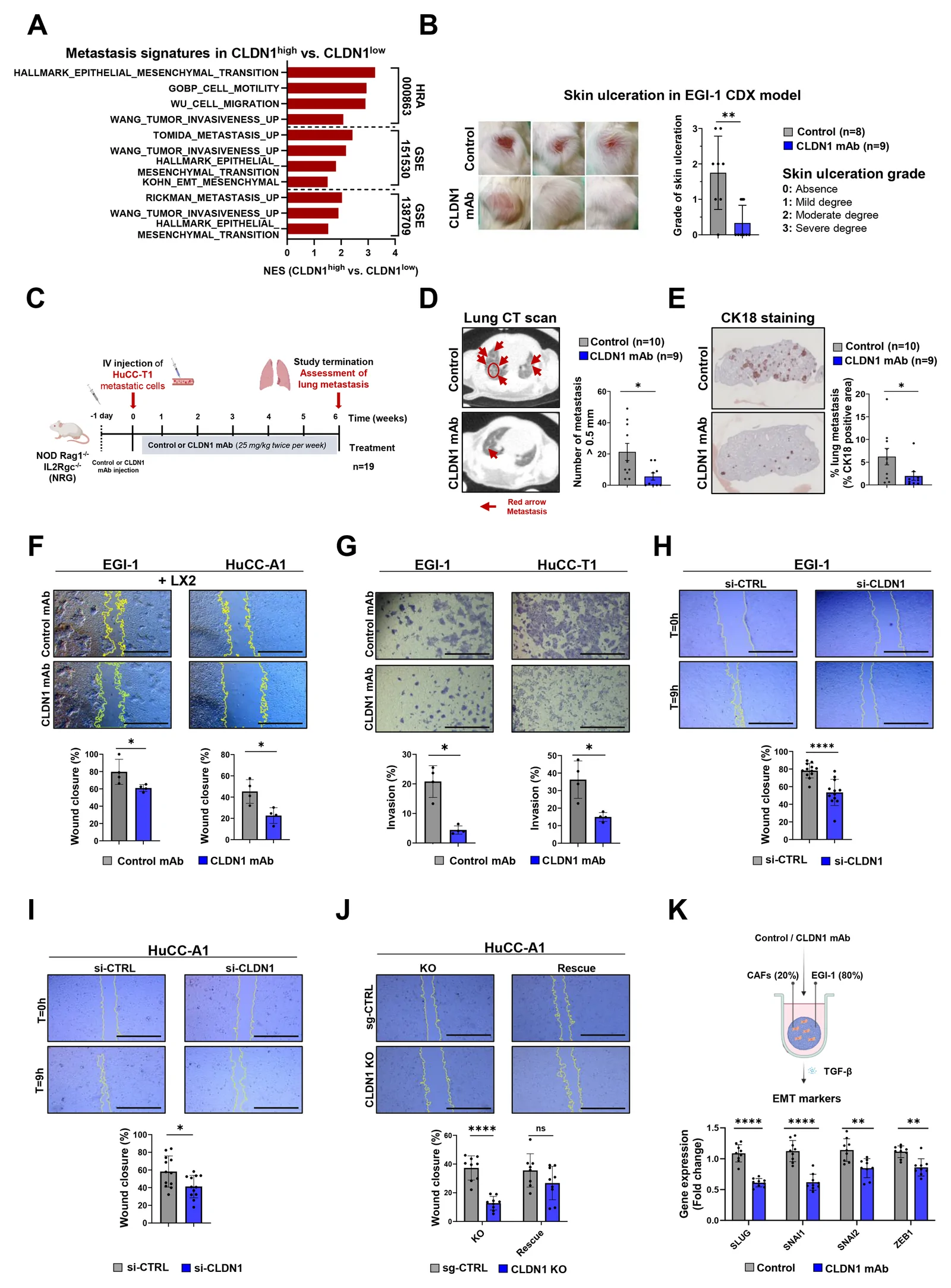
Treatment with CLDN1 mAb suppresses CCA cancer cell invasion and metastasis. **(A)** Enrichment of gene sets related to metastasis signatures in *CLDN1*^high^ compared to *CLDN1*^low^ patient tumor cells. Horizontal bars represent NES of significantly altered gene sets (FDR<0.05, Kolmogorov-Smirnov test). **(B)** Left panel, representative macroscopic images of skin ulceration in vehicle control- or CLDN1 mAb-treated groups. Right panel, quantification of the ulcerated area (*n=*8-9 mice/group, , Mann-Whitney test). Data are shown as Mean ± SD. **(C)** Illustration of the HuCC-T1 metastasis model. **(D)** Representative CT scan images and quantification of metastasis formation in vehicle control- or CLDN1 mAb-treated mice (*n=*9-10 mice/group, , Mann-Whitney test). Data are shown as Mean ± SEM. **(E)** Immunohistochemistry and quantification of CK18 expression in lung sections of vehicle control- or CLDN1 mAb-treated mice (*n=*9-10 mice/group,, Mann-Whitney test). Data are shown as Mean ± SEM. **(F)** Upper panel, representative images of the cell monolayer of EGI-1 or HuCC-A1 cells co-cultured with LX2 cells 24 h after scratching. Lower panel, percentage of closure (representative of *N=*3, , Mann-Whitney test). Scale bars, 330 µm. Data are shown as Mean ± SD. **(G)** Upper panel, representative images showing crystal violet staining visualizing invading EGI-1 or HuCC-T1 cells. Scale bars, 330 μm. Lower panel, percentage of invading cells (representative of *N=3*, *, Mann-Whitney test). Data are shown as Mean ± SD. **(H-J)** Upper panel, representative images of the cell monolayer of si-CTRL or si-CLDN1 in EGI-1 cells (H), HuCC-A1 cells (I), or CLDN1 KO and rescue HuCC-A1 cell lines (J) 9 h after scratching. Lower panel, percentage of closure (*N=*3, triplicate per condition, , Mann-Whitney test). Scale bars, 330 µm. Data are shown as Mean ± SD. **(K)** Upper panel, the experimental approach of the EGI-1 and CAFs spheroid study. Lower panel, normalized fold change gene expression of EMT gene markers in control- or CLDN1 mAb-treated spheroids (*N=*3-4, triplicate per condition, , Mann-Whitney test). Data are shown as Mean ± SD. *p<0.05, **p<0.01, ****p<0.0001

The majority of patients with CCA are ineligible for curative surgery due to the presence of metastases at the time of diagnosis, with EMT considered as a key mechanism of metastatic dissemination (37). GSEA of tissues of patients with CCA revealed a significant enrichment of metastasis signatures in CCA tumors with high *CLDN1* expression (HRA000863, GSE151530, GSE138709, FDR<0.05, Fig. 5A). Confirming patient transcriptional analyses, we found that CLDN1 mAb treatment in the EGI-1 CDX model (Fig. 4D) significantly reduced tumor-induced skin ulceration resulting from local invasion compared to control-treated mice (**p<0.01, Fig. 5B), pointing toward a therapeutic effect on tumor invasion in this specific model. To validate this observation, we used a state-of-the-art mouse model for CCA metastasis based on intravenous injection of the highly metastatic HuCC-T1 CCA cell line (Fig. 5C). Treatment with the CLDN1 mAb significantly reduced metastasis formation in lungs compared to the control group as shown by CT scan imaging (*p<0.05, Fig. 5D) and immunohistochemistry revealing a significant decrease in cytokeratin 18-positive CCA metastatic lung lesions (*p<0.05, Fig. 5E).

To understand the mechanistic events of invasion and metastasis, we performed perturbation studies in cell-based models. CLDN1 mAb treatment significantly decreased cell migration, using co-culture models including LX2 stellate cells to model the pathological desmoplastic stroma seen in CCA tumors (38) (*p<0.05, Fig. 5F). Similar results were observed on cell invasion using the transwell invasion monoculture assay (*p<0.05, Fig. 5G). The functional role of CLDN1 was confirmed by loss-of-function studies demonstrating significantly decreased cell migration in knock-down (KD) or CLDN1 KO studies (*p<0.05, ****p<0.0001, Fig 5H-J), rescued by restoration of CLDN1 expression (Fig. 5J). To understand the mechanism of action of CLDN1 mAb in suppressing metastasis, we established a model using cancer-associated fibroblasts (CAFs), as CAFs are known to promote CCA metastasis through interactive autocrine and paracrine signaling pathways (39). CAF-derived conditioned media or co-cultures induced EMT markers in EGI-1 cells (****p<0.0001, fig. S5A). Next, we assessed the effect of CLDN1 mAb treatment on EMT gene markers using spheroids composed of EGI-1 cells and CAFs to mimic a 3D growth microenvironment (40) (Fig. 5K, upper panel). Treatment with CLDN1 mAb of CCA cancer cells decreased the expression of *SLUG, ZEB1*, and *SNAI* family transcriptional repressors 1 and 2 (**p<0.01, ****p<0.0001, Fig. 5K, lower panel), shown to correlate with metastasis and poor prognosis in patients with CCA (37). Collectively, these data demonstrate that treatment with CLDN1 mAb results in a decrease of CCA metastasis and invasion, possibly through EMT inhibition of cancer cells.

### CLDN1 mediates pro-carcinogenic signaling in CCA models

Next, we aimed to elucidate the molecular mechanisms underlying CLDN1 driver role and the anti-tumoral effect of mAb treatment. RNASeq analysis in CLDN1 loss-of-function studies in vivo (Fig. 3E) as well as pharmacological targeting of CLDN1 in CDX and PDX models (Fig. 4C and F), revealed a suppression of signatures associated with cell survival, stemness and differentiation, including EMT (FDR<0.05, fig. S6A and B) across all models and interventions. Since the expression of these perturbed pathways and cancer hallmarks was already significantly suppressed in the very early phase of CLDN1 mAb treatment using the HuCC-A1 CDX model (48 hr, FDR<0.05, fig. S6C), the modulation of cancer cell fate and plasticity likely play a key role in mediating the anti-tumor efficacy of the mAb and are not secondary adaptive events that occur following long-term treatment.

**Figure 6:**
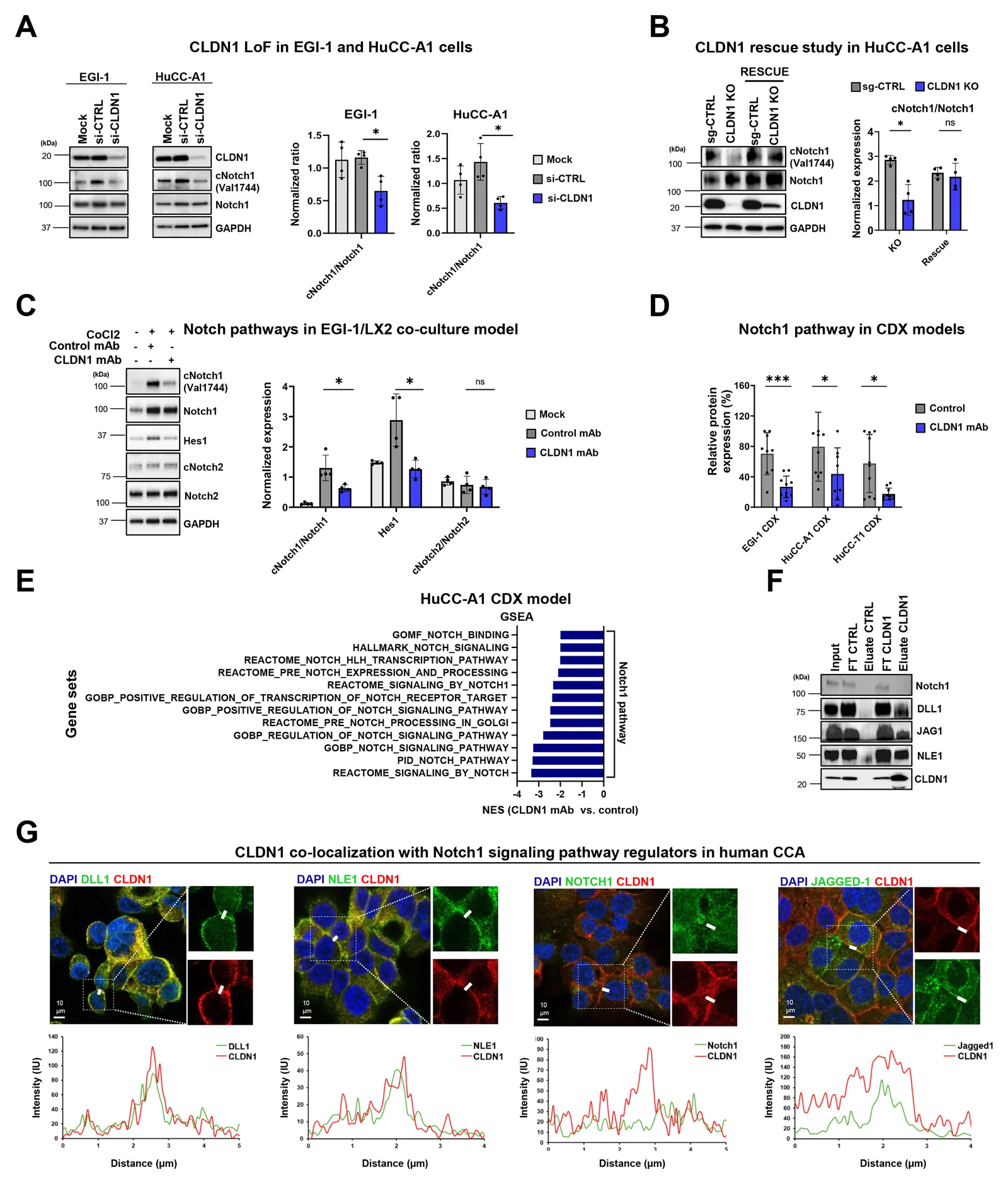
Genetic loss-of-function and pharmacological perturbation studies reveal Notch1 signaling as a key CLDN1 effector pathway. **(A)** Representative Immunoblots and normalized protein abundance of CLDN1, cleaved Notch1(cNotch1), Notch1, and GAPDH in EGI-1 and HuCC-A1 cells in mock, si-CTRL or si-CLDN1 conditions (*N*=4, , Mann-Whitney test). Data are shown as Mean ± SD. **(B)** Representative Immunoblots and normalized expression of cNotch1 (Val1744), Notch1, CLDN1 and GAPDH in HuCC-A1 CLDN1 KO or rescue cell lines (*N*=4, , Mann-Whitney test). Data are shown as Mean ± SD. **(C)** Representative Immunoblots and normalized expression of cNotch1 (Val1744), Notch1, Hes1, cNotch2, Notch2 and GAPDH in EGI-1/LX2 co-cultures stimulated with cobalt chloride (CoCl_2_) (*N*=4, Mann-Whitney test). Data are shown as Mean ± SD. **(D)** Relative protein expression of normalized ratio of cNotch1/Notch1 in EGI-1, HuCC-A1 or HuCC-T1 tumors from CDX mice (two different lysates for *n=4-*5 mice,, Mann-Whitney test). Data are shown as Mean ± SEM. **(E)** Gene set enrichment in CLDN1 compared to control mAb-treated HuCC-A1 tumors from CDX mice. Horizontal bars represent NES of significantly altered gene sets (*n=*5 mice/group, FDR<0.05, Kolmogorov-Smirnov test). **(F)** Co-immunoprecipitation of Notch1 and Notch1 ligands/regulators DLL1, JAG1 and NLE1 with CLDN1 in the cell membrane of HuCC-A1 cells (*N=*3). **(G)** Representative high-resolution z-stack confocal images showing HuCCA-1 cells stained for DAPI (blue) CLDN1 (red-AF647) and Notch1 and Notch1 ligands/regulators DLL1, NLE1 and JAG1 (green-AF488) using ZEISS LSM 800 with a Plan-Apochromat 63x / 1.4 oil immersion objective (*N=*3). For DLL1, NLE1 and JAG1 staining, HuCC-A1 cells were treated for 24 h with 100 µM of cobalt chloride prior to staining. Fluorescence intensity profile plots were generated using ImageJ. Scale bars, 10 µm. *p<0.05, **p<0.01, ***p<0.001

Given that both genetic CLDN1 loss-of-function (Fig. 3C and E) and mAb perturbation studies (fig. S6A to C) altered cancer stemness, invasion, and metastasis signatures across models, we examined the signaling pathways underlying these hallmarks of cancer. Since Notch1 signaling has been identified as a key pathway regulating cancer cell differentiation, proliferation, invasion and metastasis (41), we investigated its functional role in CLDN1-mediated carcinogenesis. Indeed, CLDN1 loss-of-function studies using CLDN1 KD and KO models significantly reduced Notch1 signaling (*p<0.05, Fig. 6A and B, fig. S10D and E). In addition, a rescue study in the HuCC-A1 CLDN1 KO cells where CLDN1 re-expression restored Notch1 signaling (*p<0.05; Fig. 6B, fig. S10E) provides functional proof of the Notch1 signaling pathway as a CLDN1 effector. Similarly, CLDN1 mAb treatment selectively suppressed Notch1 signaling across all models (*p<0.05, ***p<0.001, Fig. 6C and D, fig. S7A and B, and fig. S10F). Transcriptomic analyses confirmed the downregulation of Notch1-associated signaling pathways (FDR<0.05, Fig. 6E) and Notch1 target genes (fig. S6D). Next, we investigated the molecular mechanisms initiating CLDN1-mediated Notch1 signaling through CLDN1 co-immunoprecipitation and confocal co-localization studies at the cell membrane of HuCC-A1 cells. Our data identified the Notch1 regulators Delta-like protein 1 (DLL1), Notchless homologue 1 (NLE1), and Jagged-1 (JAG1) (Fig. 6F and G, fig. S10G) as CLDN1-interacting partners, suggesting that CLDN1 engages with canonical Notch1 regulators at the CCA cell membrane to induce Notch1 signaling.

Next, we explored additional signaling pathways implicated in cancer stemness, invasion and metastasis (2). We identified TROP2 and its downstream effector STAT3, as well as YAP1 and its upstream regulator LATS1 as additional stemness and invasion candidate mediators implicated in CLDN1-driven CCA pathogenesis. Both CLDN1 mAb perturbation and CLDN1 loss-of-function studies significantly reduced YAP1/LATS1 and TROP2/STAT3 signaling in cell-based and CDX models (*p<0.05, fig. S7C to F, fig. S8A to C). Moreover, CLDN1 co-immunoprecipitation and confocal co-localization studies identified TROP2 as a CLDN1 interactant at the cell membrane further confirming a mechanistic role of this pathway (fig. S8D and E).

In summary, our findings suggest that CLDN1 acts as an oncogenic driver by perturbing Notch1 signaling through Notch1 ligands-CLDN1 interaction at the cancer cell membrane. In addition, TROP2/STAT3 and YAP1/LATS1 signaling may contribute to CLDN1 mediated perturbation of cancer cell stemness and enhancement of tumor proliferation, invasion, and metastasis.

### Treatment with CLDN1 mAb alters cancer cell plasticity, inhibits oncogenic signaling and reverts chemotherapy resistance gene expression in patient-derived organoids

**Figure 7:**
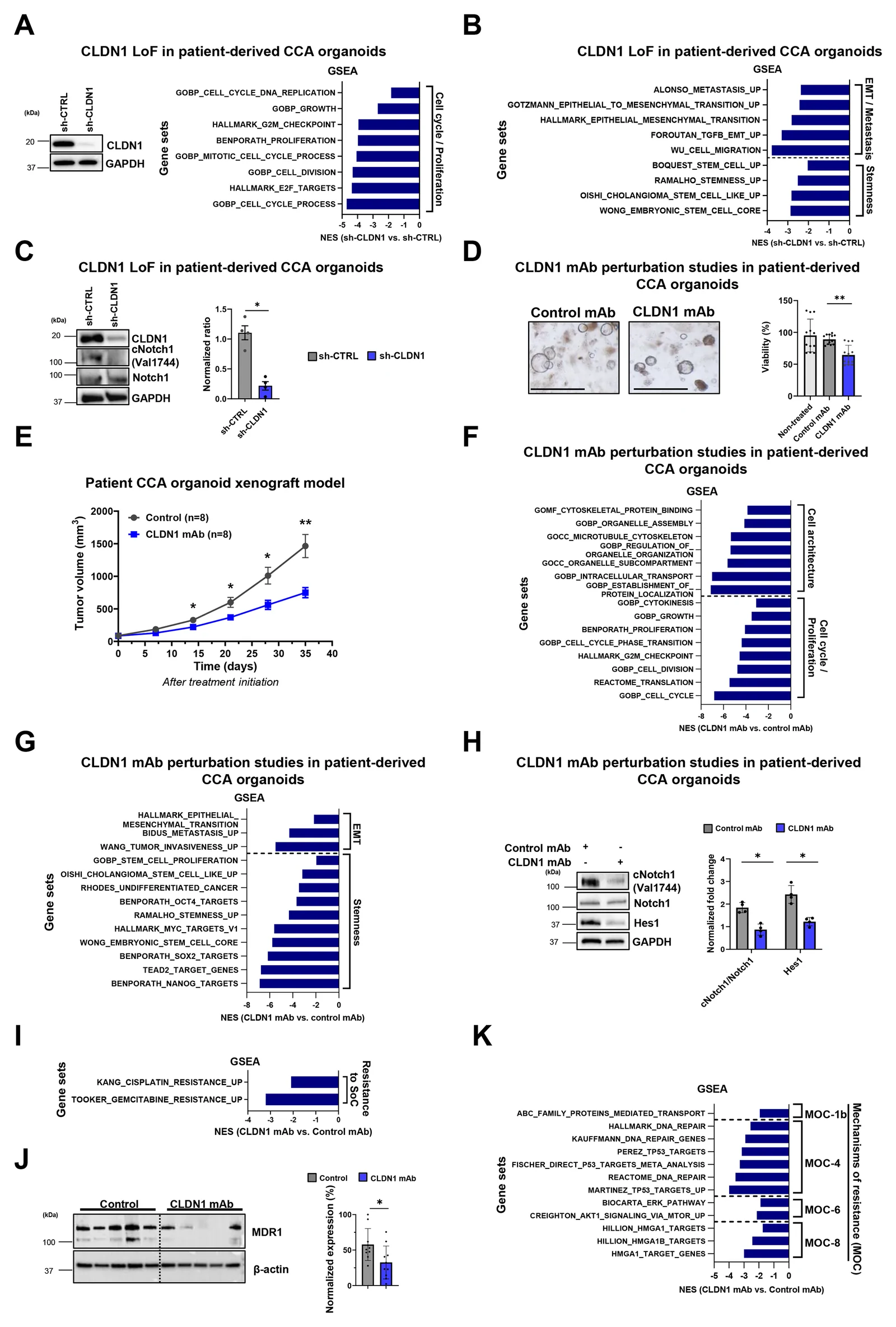
Treatment with CLDN1 mAb alters cancer cell plasticity and inhibits oncogenic signaling in patient-derived organoids. **(A)** Left panel, representative Immunoblot of CLDN1 and GADPH expression in shCTRL or shCLDN1 transduced organoids (*N=3*). Right panel, RNASeq gene set enrichment sh-CLDN1 compared to sh-CTRL transduced organoids. Horizontal bars represent NES of significantly altered gene sets (*N=*3/group, FDR<0.05, Kolmogorov-Smirnov test). **(B)** RNASeq gene set enrichment of sh-CLDN1 compared to sh-CTRL organoids. Horizontal bars represent NES of significantly altered gene sets (*N=*3/group, FDR<0.05, Kolmogorov-Smirnov test). **(C)** Representative Immunoblots and normalized expression of CLDN1, cNotch1 (Val1744), Notch1, and GAPDH in sh-CTRL or sh-CLDN1 organoids (*N*=4,, Mann-Whitney test). Data are shown as Mean ± SD. **(D)** Organoid perturbation studies. Left panel, representative images of commercially available human CCA-derived primary organoids after 7 days of treatment with the isotype control mAb or CLDN1 mAbs. Scale bars, 330 µm. Right panel, percentage of cell viability (*N=*3, quadruplicate per condition, , Mann-Whitney test). Data are shown as Mean ± SD. **(E)** In vivo validation. Tumor growth in vehicle control- or CLDN1 mAb-treated mice in a patient-derived CCA organoid xenograft model (*n=*8 mice/group,, Mann-Whitney test). Data are shown as Mean ± SEM. **(F)** Gene set enrichment in CLDN1 mAb- compared to isotype control mAb-treated organoids. Horizontal bars represent NES of significantly altered gene sets (*n=*3/group, FDR<0.05, Kolmogorov-Smirnov test). **(G)** Gene set enrichment in CLDN1 mAb-treated compared to isotype control mAb-treated organoids. Horizontal bars represent NES of significantly altered gene sets (*n=*3/group, FDR<0.05, Kolmogorov-Smirnov test). **(H)** Representative Immunoblots and normalized expression of cNotch1 (Val1744), Notch1, Hes1, and GAPDH in CCA organoids treated with isotype control- or CLDN1 mAbs (*N*=4, , Mann-Whitney test). Data are shown as Mean ± SD. **(I)** Effect of the CLDN1 mAb on expression of cisplatin and gemcitabine resistance marker genes compared to isotype control mAb in patient-derived organoids. Horizontal bars represent NES of significantly altered gene sets (FDR<0.05, Kolmogorov-Smirnov test). **(J)** Representative Immunoblots and normalized expression of MDR1 and β-actin in tumors derived from the EGI-1 CDX model treated with CLDN1 mAbs or control mAb (two different lysates for *n=*5 mice,, Mann-Whitney test). Data are shown as Mean ± SEM. **(K)** Enrichment of gene sets predicting resistance to chemotherapy in CLDN1 mAb-versus isotype control mAb-treated patient-derived organoids. Horizontal bars represent NES of significantly altered gene sets (*n=*3/group, FDR<0.05, Kolmogorov-Smirnov test). *p<0.05, **p<0.01

To validate the functional role of CLDN1 in cancer cell fate and signaling in models of patients with CCA, we conducted loss-of-function studies in organoids established from tissues of patients with CCA (Fig. 7A, left panel, fig. S10H). As observed in cell-based and CDX models, gene expression analyses revealed a downregulation of the expression of gene sets encoding for proliferation, stemness, EMT and metastasis (FDR<0.05, Fig. 7A and B, fig. S9A). In addition, genetic CLDN1 loss-of-function significantly reduced Notch1 signaling confirming again the functional relevance of this pathway as CLDN1 effector in patient-derived organoids (*p<0.05, Fig. 7C, fig. S10I).

The functional role of CLDN1 in CCA organoid cancer biology was again validated by perturbation studies. CLDN1 mAb treatment modulated the phenotype of the organoids with an altered morphology reflected by the appearance of smaller and condensed structures, in contrast to well-circumscribed, round organoids in the control mAb-treated group (Fig. 7D, left panel). This phenotypic change was accompanied by a significant decrease in viability upon treatment with the CLDN1 mAb (**p<0.01, Fig. 7D, right panel). The effect of CLDN1 on organoid viability was confirmed in vivo in a CCA organoid-based xenograft mouse model where treatment with CLDN1 mAb significantly reduced tumor growth (*p<0.05, **p<0.01, Fig. 7E, fig. S9B).

In line with the observed decrease in organoid viability, proliferation and morphological changes, transcriptomic profiling of CLDN1 mAb-treated organoids revealed a pronounced effect on cell architecture and proliferation (FDR<0.05, Fig. 7F), and a similar downregulation of GSEA signatures encoding for oncogenic signaling, metabolism, EMT and stemness (FDR<0.05, Fig. 7G, fig. S9C). Moreover, and in addition to the suppression of CCA stemness signatures, CLDN1 mAb treatment also downregulated signature targets related to SOX2, OCT4, and MYC, also known as the Yamanaka factors (42) responsible for reprogramming differentiated cells toward induced pluripotent stem cells (FDR<0.05, Fig. 7G). We further confirmed the downregulation of key signaling pathways implicated in CCA stemness, including the Notch1 pathway upon treatment with the CLDN1 mAb (*p<0.05, Fig. 7H, fig. S9C, and fig. S10J) on both RNA and protein levels in CCA organoids treated with isotype control or CLDN1 mAbs. Collectively, these data confirm results obtained from cell lines, CDX and PDX models (Figs. 3-6) in organoids (Fig. 7), a patient-derived model shown to be highly predictive of the translation of preclinical proof-of-concept into the clinic.

Lasty, the clinical outcome of patients with CCA treated with cisplatin and gemcitabine-based chemotherapy remains dismal, mainly due to intrinsic or acquired chemoresistance (2). Treatment with the CLDN1 mAb suppressed signatures related to cisplatin and gemcitabine resistance (FDR<0.05, Fig. 7I). To elucidate the potential mechanisms by which the CLDN1 mAb might overcome chemoresistance, we further dissected the mechanisms of chemoresistance (MOC) that define the CCA resistome (2). Beside the downregulation of the expression of multi-drug resistance 1 (MDR1), a membrane receptor involved in cancer metastasis and chemotherapy resistance (43) (*p<0.05, Fig. 7K) confirmed by the downregulation of MOC-1b signature, treatment with the CLDN1 mAb suppressed the expression of signatures related to MOC-4, -6 and -8 (FDR<0.05, Fig. 7J). Since at the same time CLDN1 expression is upregulated upon cisplatin or gemcitabine treatment (*p<0.05, ****p<0.0001, fig. S9D), these data provide a rationale for combining CLDN1 mAbs with the SoC chemotherapy.

## Discussion

Here, we uncovered CLDN1 as a previously unknown driver and therapeutic target for extrahepatic and intrahepatic CCA (fig. s11). We discovered CLDN1 as a CCA driver using gain- and loss-of-function studies in cell-based and mouse models (Figs. 1-6). Spatial transcriptomics and mechanistic studies revealed a functional role of cancer cell membrane-exposed CLDN1 as a signal transducer of multiple pro-tumorigenic signaling cascades which regulate cell plasticity and fate, stemness and EMT (Figs. 2 and 6). Targeting exposed CLDN1 on the cancer cell surface using highly specific mAbs showed a robust suppression of tumor growth, invasion and metastasis across a large series of different pre-clinical and patient-derived models including PDX and organoids (Figs. 4, 5, 7).

Our data are consistent with the following mechanistic model (fig. s11): CLDN1 expressed on the CCA cancer cell surface interacts with Notch1 ligands/regulators DLL1, JAG1 and NLE. This interaction at the cancer cell membrane likely induces oncogenic signaling of the Notch1 (Fig. 6) mediating modulation of cell plasticity and stemness and resulting in enhanced tumor proliferation, invasion and metastasis (Figs. 3J, 5D-J). Whereas TROP2/STAT3 and YAP1/LATS1 were identified as additional signaling candidate pathways, the marked and early dysregulation across models combined with co-IP and rescue studies (Figs. 6, 7), suggest that the Notch1 pathway is likely a predominant signaling hub.

In contrast to the high toxicity of the SoC, CLDN1 mAbs have been shown to exhibit an excellent safety profile, without detectable major adverse effects as shown in the current and previous studies, including in non-human primates (16, 18), as well as in healthy volunteers (44). The absent toxicity is likely due to the high specificity of the mAbs which target only exposed non-junctional CLDN1 outside the tight junctions and therefore do not affect the epithelial barrier function (16, 18, 33, 45).

Our study revealed that *CLDN1* expression is positively correlated with actionable genomic alterations (e.g., IDH1) for which targeted therapies have been approved (Fig. 1) and that treatment with highly specific CLDN1 mAbs reduces expression of genes and pathways mediating resistance to platinum-based SoC in patient organoids (Fig. 7I to K) and tumors in vivo (Fig. 7J). These findings provide a clear rationale for CCA treatment with CLDN1 mAbs both as a single agent as well as in combination therapy either with SoC or targeted therapies to overcome the multidrug intrinsic or acquired resistant phenotype in CCA.

Key strengths of our study are the use of highly translatable patient models, genetic validation, the absence of detectable toxicity, as well as the consistency of results across a large series of complementary model systems. The study has the following limitations: First, we cannot exclude that other signaling pathways or additional mechanistic events are at play mediating the anti-cancer effect of the antibodies. Second, future studies will be needed to delineate the molecular phenotype of CCA tumors predicting response and resistance to CLDN1 mAb treatment.

Collectively, these results unravel CLDN1 as a previously undiscovered therapeutic CCA target and provide an opportunity for the clinical development of CLDN1-specific monoclonal antibodies for the treatment of CCA to improve the dismal outcome of patients.

## Materials and Methods

### Study design

Computational transcriptomic analyses were conducted in publicly available and own patient cohorts of CCA. Target expression was characterized by immunohistochemistry and immunofluorescence in 170 healthy and CCA tissues of different patient cohorts. In vivo hydrodynamic tail vein injection and CDX loss-of-function studies were performed to validate the role of CLDN1 as a driver of CCA. Targeting membrane-exposed CLDN1 using highly specific mAbs was evaluated as a therapeutic modality to treat CCA in a large panel of different in vivo and ex vivo models. The molecular mechanism of action was studied by co-immunoprecipitation studies, single-cell and bulk transcriptomic as well as proteomic analyses in cell-based and in vivo models of CCA, including patient-derived 3D organoids. Experiments were partially blinded and performed in triplicate in at least three independent experiments, unless otherwise stated.

### Patient-derived tissues and cells

Human CCA tissue samples were obtained from patients who had undergone tumor resections for cholangiocellular carcinoma at Strasbourg University Hospitals, France and Groupe Hospitaler Saint Vincent, Strasbourg (DC-2016-2616 and RIPH2 LivMod IDRCB 2019-A00738-49, ClinicalTrial NCT04690972). All patients provided written informed consent, and the protocol followed the ethical principles of the Declaration of Helsinki and was approved by local independent ethics committees. Sampling of human liver tissue for immunostaining was conducted by US Biomax Inc. with informed consent of all donors and under HIPPA approved protocols. For CLDN1 expression analysis, CCA tissues were obtained from local biobanks of the Department of Imaging and Pathology, KU Leuven and the Biological Resource Centre (CRB) of Strasbourg University Hospitals, France. Protocols were approved by the local Ethics Committee of the University of Strasbourg Hospitals. All material was collected during a medical procedure strictly performed within the frame of the medical treatment of the patient. Informed consent was provided according to the Declaration of Helsinki. Detailed patient information and informed consent procedures were implemented by the CRB of Strasbourg University Hospitals. Demographic data and clinical characteristics of enrolled patients are summarized in tables S1-3, 5.

### Human CCA cell lines

EGI-1 cells were obtained from the Leibniz-Institute-DSMZ-German-Collection of Microorganisms and Cell Cultures, Germany. EGI-1 cells have been described in Vijay *et al.* (46). HuCC-A1, HuCC-T1 cells were obtained from the Japanese-Collection of Research Bioresources (JCRB) (JCRB0425 and JCRB1657) through FUJIFILM Wako Europe. EGI-1, HuCC-A1 and HuCC-T1 cells were cultured in RPMI 1640 medium supplemented with 10% fetal bovine serum (FBS), 1% non-essential amino acids (Gibco) and 0.5% gentamicin (Gibco). All cells were maintained in a humidified incubator at 37 °C and 5% CO_2_. Cells between passage 3 and 20 were used for all experiments. All our cell lines were tested and certified *Mycoplasma* negative.

### CLDN1 gain-of-function studies using hydrodynamic tail vein injections

Experiments were conducted in accordance with the guidelines of the German Animal Protection Law and were approved by local committees of government of the Baden-Württemberg (license number 35-9185.81/G-275/18). The tail vein of an 8-week-old C57BL/6J male mouse was dilated by heating under red light, and a 2 ml saline solution containing 25 µg KRAS^G12D^, 60 µg p19^ARF^, 7 µg sleeping beauty, and 25 µg pT3-Empty or pT3-CLDN1 plasmids for the KRAS/p19 model or 10 µg YAP^S127^, 5 µg myr-AKT, 5 µg sleeping beauty, and 25 µg pT3-Empty or pT3-CLDN1 plasmids for the AKT/YAP model were injected within 4-8 seconds at a constant rate. 6 mice were included in each group. Mice were observed daily for any signs of abnormality and sacrificed once the termination criteria were met.

### CLDN1-specific antibodies

Humanized CLDN1-specific mAb H3L3 IgG4 has been described (33). The isotype control antibody used in this study was motavizumab (47). It targets the conserved antigenic site A on the fusion (F) glycoprotein of the respiratory syncytial virus.

### CCA patient-derived liver organoids

Organoid establishment and expansion. Organoid model HCM-CSHL-0433-C24 (PDM-217) was purchased from ATCC. Organoids were re-suspended in 50 µl Cultrex Basement Membrane Extract (BME) Type 2 (3532-005-02, R&D) and seeded as domes onto 24 well plates. Organoids were expanded as previously described (16) and organoids of passage 3 were used for perturbation studies. Organoid perturbation study. Organoid perturbation studies were performed as described previously (16), with media and treatment being renewed 3 days after treatment initiation. At day 7, representative microscopic images were taken, and organoids were harvested for RNA extraction using PicoPure RNA Isolation Kit (KIT0204, Applied Biosystems) according to the manufacturer’s instructions. The same treatment protocol was followed for signaling studies, with 1 full organoid well being expanded into eight wells for optimal seeding density before treatment. Viability assay. Organoids were seeded in 96 well plates pre-coated with BME domes and treated with 10 µg/ml isotype control mAb or CLDN1 mAb. On day 7 post-treatment, viability was assessed using CellTiterGlo 3D (Promega) according to the manufacturer’s instructions.

### Sstatistical analysis

All data were compared using Students’ t-test, when normally distributed or non-parametric tests (Mann-Whitney test, Kolmogorov-Smirnov test, log-rank Mantel-Cox, one-way ANOVA) (GraphPad Prism v10.0). Results with a p-value <0.05 or an FDR-value <0.05 were considered statistically significant. *p<0.05, **p<0.01, ***p<0.001, and ****p<0.0001. Graphs were generated using GraphPad Prism v10.0. All individual level data for n<20 are presented in data file S1.

## Supplementary Material

Supplementary Checklist

Supplementary Data file S1

Supplementary Table S1

## Data Availability

All data associated with this study are available in the main text or the Supplementary Materials. Transcriptomic data reported in this paper has been deposited at the Gene Expression Omnibus (GEO) database with the accession number GSE262166. HuCC-T1 and HuCC-A1 cells are available from the Japanese Collection of Research Bioresources (JRCB), and HCM-CSHL-0433-C24 (PDM-217) organoids are available from ATCC and were obtained through material transfer agreements of JRCB and ATCC with the University of Strasbourg and Inserm. All materials used or generated in this study are commercially available or will be supplied upon reasonable request.

## One Sentence Summary

Exposed Claudin-1 is a mediator and therapeutic target for cholangiocarcinoma.
