# Supplementary material for: Claudin-1 targeting suppresses tumor growth, invasion and metastasis in patient-derived cholangiocarcinoma models
Source: Sci Transl Med. Author manuscript; Available in PMC 2026 Oct 15. (PMC7619551; doi:10.1126/scitranslmed.adp2595)
Supplement: Supplementary file 1 [file EMS216145-supplement-Supplementary_Checklist.pdf]

Materials Design Analysis Reporting (MDAR)
Checklist for Authors

The MDAR framework establishes a minimum set of requirements in transparent reporting applicable to studies in the life sciences (see Statement of Task: doi:10.31222/osf.io/9sm4x.). The MDAR checklist is a tool for authors, editors, and others seeking to adopt the MDAR framework for transparent reporting in manuscripts and other outputs. Please refer to the MDAR Elaboration Document for additional context for the MDAR framework.

For all that apply, please note where in the manuscript the required information is provided.

Materials:

Newly created materials	indicate where provided: page no/section/legend)	n/a
The manuscript includes a dedicated "materials availability statement" providing transparent disclosure about availability of newly created materials including details on how materials can be accessed and describing any restrictions on access.	All materials used in this paper are commercially available as indicated in the manuscript	

Antibodies	indicate where provided: page no/section/legend)	n/a
For commercial reagents, provide supplier name, catalogue number and RRID, if available.	All antibodies used in this studies and their providers are listed in supplementary materials and methods, western blot section	

DNA and RNA sequences	indicate where provided: page no/section/legend)	n/a
**Short novel DNA or RNA including primers, probes:** Sequences should be included or deposited in a public repository.	Sequances are listed in Supplementary materials and methods, CLDN1 knock-out (KO) using CRISPR-Cas9 technology and rescue study section and gene expression analyses in 2D cell culture experiments section.	

Cell materials	indicate where provided: page no/section/legend	n/a
**Cell lines:** Provide species information, strain. Provide accession number in repository **OR** supplier name, catalog number, clone number, **OR** RRID.	All related information is listed in main manuscript, Materials and methods section, Human CCA cell lines section. EGI-1 cells were obtained from the Leibniz-Institute-DSMZ-German-Collection of Microorganisms and Cell Cultures, Germany. HuCC-A1, HuCC-T1 cells were obtained from the Japanese-Collection of Research Bioresources (JCRB) (JCRB0425 and JCRB1657) through FUJIFILM Wako Europe. Organoid model HCM-CSHL-0433-C24 (PDM-217) was purchased from ATCC.	
**Primary cultures:** Provide species, strain, sex of origin, genetic modification status.		X

Experimental animals	indicate where provided: page no/section/legend)	n/a
**Laboratory animals or Model organisms:** Provide species, strain, sex, age, genetic modification status. Provide accession number in repository **OR** supplier name, catalog number, clone number, **OR** RRID.	For *in vivo* experiments using cell line-dervied or patient-derived xenografts, non-Obese Diabetic Rag1$^{-/-}$ IL2Rgc$^{-/-}$ (NRG) were used. For gain-of-function studies using immunocompetent mice, C57BL/6J mouse were used. For loss-of-function and gain-of-function studies, male mice were used. For CLDN1 mAb perturbation studies, male and female mice were used unless otherwise indicated. For all experiments, mice aged 8 weeks old were used.	
**Animal observed in or captured from the field:** Provide species, sex, and age where possible.		X

Plants and microbes	indicate where provided: page no/section/legend)	n/a
**Plants:** provide species and strain, ecotype and cultivar where relevant, unique accession number if available, and source (including location for collected wild specimens).		X
**Microbes:** provide species and strain, unique accession number if available, and source.		X

Human research participants	indicate where provided: page no/section/legend) or	n/a

	state if these demographics were not collected	
If collected and within the bounds of privacy constraints report on age, sex and gender or ethnicity for all study participants.	Supplementary files, supplementary tables S1, S2, S4 and S5 provide clinical and histopathological information on the patient tissues used. Under each table, the following is indicated: due to patient data protection regulations, sex and age of patients are not shown but are available from the corresponding author upon reasonable request.	

Design:

Study protocol	indicate where provided: page no/section/legend)	n/a
If study protocol has been pre-registered, provide DOI. For clinical trials, provide the trial registration number **OR** cite DOI.		X

Laboratory protocol	indicate where provided: page no/section/legend)	n/a
Provide DOI **OR** other citation details if detailed step-by-step protocols are available.		X

Experimental study design (statistics details)		
**For in vivo studies:** State whether and how the following have been done	**indicate where provided: page no/section/legend. If it could have been done, but was not, write not done**	**n/a**
Sample size determination	The sample size was calculated using the website http://powerandsamplesize.com/, based on a comparison of means between two groups of equal size, with a statistical power of 80% and an alpha risk of 5%.	
Randomisation	Mice were randomized based on the tumor size. When the average of tumor volumes reached 50-100 mm^3, mice were randomized into 2 groups in a way that the tumor size at the beginning of the treatment was comparable between the control and the treatment groups.	
Blinding		X
Inclusion/exclusion criteria	When the average of tumor volumes reached 50-100 mm^3, mice were randomized and included in the study protocol. Mice were planned to be excluded from the protocol if they met the ethical endpoint based on the general appearance, tumor size, weight…	

Sample definition and in-laboratory replication	indicate where provided: page no/section/legend	n/a
State number of times the experiment was replicated in laboratory.	As indicated in the materials and methods section, study design section, experiments were performed in triplicate in at least three independent experiments, unless otherwise stated.	
Define whether data describe technical or biological replicates.	Each experiment was repeated at least 3 independent times (biological replicates) in triplictaes (technical replicates)	

Ethics	indicate where provided: page no/section/legend	n/a
**Studies involving human participants:** State details of authority granting ethics approval (IRB or equivalent committee(s), provide reference number for approval.	As indicated in the materials and methods section, patient-derived tissues and cells section, human CCA tissue samples were obtained from Strasbourg University Hospitals, France and Groupe Hospitaler Saint Vincent, Strasbourg (DC-2016-2616 and RIPH2 LivMod IDRCB 2019-A00738-49, ClinicalTrial NCT04690972). All patients provided written informed consent, and the protocol followed the ethical principles of the Declaration of Helsinki and was	

	approved by local independent ethics committees. Sampling of human liver tissue for immunostaining was conducted by US Biomax Inc. with informed consent of all donors and under HIPPA approved protocols. For CLDN1 expression analysis, CCA tissues were obtained from local biobanks of the Department of Imaging and Pathology, KU Leuven and the Biological Resource Centre (CRB) of Strasbourg University Hospitals, France. Protocols were approved by the local Ethics Committee of the University of Strasbourg Hospitals. All material was collected during a medical procedure strictly performed within the frame of the medical treatment of the patient. Informed consent was provided according to the Declaration of Helsinki.	
**Studies involving experimental animals:** State details of authority granting ethics approval (IRB or equivalent committee(s), provide reference number for approval.	As indicated in the materials and methods section, CLDN1 gain-of-function studies using hydrodynamic tail vein injections section, Experiments were conducted in accordance with the guidelines of the German Animal Protection Law and were approved by local committees of government of the Baden-Württemberg (license number 35-9185.81/G-275/18). As indicated in the supplementary materials and methods section, CDX and PDX mouse models, All perturbation experiments were performed at the animal facility of Inserm U1110 (approval number E67-482-7), and CLDN1 loss-of-function study was performed at the CRBS Platform for Functional Exploration and Experimental Resources (approval number G67-482-35) according to local laws, ethics committee approval, and authorization by the French Ministry of Research and Higher Education (approval number APAFIS #27709-202010151425640 4 v4, #50417-202407161436430 v5, #22327-2019100815074277 v3, #35280-2022020810339017 v3, #35040-2022013107151931 v5)	
**Studies involving specimen and field samples:** State if relevant permits obtained, provide details of authority approving study; if none were required, explain why.	As indicated in the materials and methods section, patient-derived tissues and cells section, human CCA tissue samples were obtained from Strasbourg University Hospitals, France and Groupe Hospitaler Saint Vincent, Strasbourg (DC-2016-2616 and RIPH2 LivMod IDRCB 2019-A00738-49, ClinicalTrial NCT04690972). All patients provided written informed consent, and the protocol followed the ethical principles of the Declaration of Helsinki and was approved by local independent ethics committees. Sampling of human liver tissue for immunostaining was conducted by US Biomax Inc. with informed consent of all donors and under HIPPA approved protocols. For CLDN1 expression analysis, CCA tissues were obtained from local biobanks of the Department of Imaging and Pathology, KU Leuven and the Biological Resource Centre (CRB) of Strasbourg University Hospitals, France. Protocols were approved by the local Ethics Committee of the University of Strasbourg Hospitals. All material was collected during a medical procedure strictly performed within the frame of the medical treatment of the patient. Informed consent was provided according to the Declaration of Helsinki.	

Dual Use Research of Concern (DURC)	indicate where provided: page no/section/legend	n/a
If study is subject to dual use research of concern regulations, state the authority granting approval and reference number for the regulatory approval.		X

Analysis:

Attrition	indicate where provided: page no/section/legend	n/a
Describe whether exclusion criteria were preestablished. Report if sample or data points were omitted from analysis. If yes report if this was due to attrition or intentional exclusion and provide justification.		X

Statistics	indicate where provided: page no/section/legend	n/a
Describe statistical tests used and justify choice of tests.	Main manuscript, Data acquisition and statistical analysis. All data were compared using Students' t-test, when normally distributed or non-parametric tests (Mann-Whitney test, Kolmogorov-Smirnov test, log-rank Mantel-Cox, one-way ANOVA) (GraphPad Prism v10.0). Results with a p-value <0.05 or an FDR-value <0.05 were considered statistically significant. *p<0.05, **p<0.01, ***p<0.001, and ****p<0.0001. All individual level data for n<20 are presented in data file S1.	

Data availability	indicate where provided: page no/section/legend	n/a
For newly created and reused datasets, the manuscript includes a data availability statement that provides details for access or notes restrictions on access.	This is indicated in materials and mrthods section, data and materials availability section.	
If newly created datasets are publicly available, provide accession number in repository **OR** DOI **OR** URL and licensing details where available.	Transcriptomic data reported in this paper has been deposited at the Gene Expression Omnibus (GEO) database with the accession number GSE262166.	
If reused data is publicly available provide accession number in repository **OR** DOI **OR** URL, **OR** citation.	Publicaly available datasets used in this manuscript are: GSE32225, GSE132305, GSE107943, GSE89747, GSE89748, GSE138709, GSE138709, GSE151530 and HRA000863.	

Code availability	indicate where provided: page no/section/legend	n/a
For all newly generated custom computer code/software/mathematical algorithm or re-used code essential for replicating the main findings of the study, the manuscript includes a data availability statement that provides details for access or notes restrictions.		X
If newly generated code is publicly available, provide accession number in repository, **OR** DOI **OR** URL and licensing details where available. State any restrictions on code availability or accessibility.		X
If reused code is publicly available provide accession number in repository **OR** DOI **OR** URL, **OR** citation.		X

Reporting

MDAR framework recommends adoption of discipline-specific guidelines, established and endorsed through community initiatives. Journals have their own policy about requiring specific guidelines and recommendations to complement MDAR.

Adherence to community standards	indicate where provided: page no/section/legend	n/a
State if relevant guidelines (e.g., ICMJE, MIBBI, ARRIVE) have been followed, and whether a checklist (e.g., CONSORT, PRISMA, ARRIVE) is provided with the manuscript.		X
